# What Is Required for Edible Insects to Become Medical Food? From a Health Professionals and Caregivers’ Perspective

**DOI:** 10.3390/insects11060388

**Published:** 2020-06-23

**Authors:** Harry Jeong, Kwangsoo Shin

**Affiliations:** Department of Biomedical Convergence, College of Medicine, Chungbuk National University, Chungdae-ro 1, Seowon-gu, Cheong-ju, Chungbuk 28644, Korea; harry@g.cbnu.ac.kr

**Keywords:** questionnaire survey, alternative, novel food, entomophagy, promotion strategy

## Abstract

The challenge in the edible insect industry is to reverse consumers’ aversion to insects, which is a barrier to their consumption. This requires innovation by users rather than producers. This study aimed to present how edible insects could be promoted as medical foods from the health professionals and caregivers’ perspective. By analyzing the characteristics of the medical foods market, this study found a niche market and plan to develop medical foods using edible insects as an alternative to meet the needs of consumers. The survey participants were caregivers, nurses, and doctors as providers of medical foods. Based on the survey results, this study proposed strategies to reduce consumers’ aversion to edible insects and increase their consumption. To promote insect medical foods, it is required to hold frequent insect-related events and use clean raw materials.

## 1. Introduction

The recent increase in interest in edible insects has led to the development of various processed foods based on insects. This creates bright prospects for the growth of the food industry. While entomophagy is not common in Korea, European and American consumers are generally not familiar with insect-based processed foods. Korea did use grasshoppers and crickets as food sources during past agricultural periods. However, as industrialization progressed and the number of generations lacking exposure to edible insects increased, the refusal to eat insects has increased [1]. Since 2016, edible insects have been legally registered as a new type of food in Korea. People have high expectations in terms of the health benefits offered by edible insects, while some use them for medicinal purposes [2]. Insects themselves are raw materials that are hardly influenced by the genetically modified organisms (GMOs), pesticides, and antibiotics that users are concerned about. Additionally, as it is easier to control insects’ breeding conditions than those of animals and plants. They can be produced more safely [3]. A medical food is defined as a food product administered enterally under the supervision of a physician for the dietary management of a specific disease, disorder, or medical condition [4]. Many patients who use medical foods expect to consume nutrients as well as various bioregulatory components within the context of a limited diet. Researchers are developing a variety of medical foods with health benefits. 

The interest in edible insects has increased since the Food and Agriculture Organization of the United Nations (FAO) referred to insects as food for humans [3]. Insects have been institutionally accepted as food in many regions and have been consumed for a long time [5]. In Asian, South American, African, and European primitive cultures, a variety of insect species were consumed [2,6]. DeFoliart [7] and Yen [8] studied the use of insects as food. Williams and Williams [9] studied aquatic insects and their potential to contribute to the human diet. Yun and Hwang [10] developed more than 100 general food recipes and more than 50 recipes for patients. In addition, because of the potential of protein-rich edible insects, they are being studied as patient meals to restore the health of patients, including cancer patients [11].

Medical foods can be classified as general nutritional supplements and customized supplements for specific disease groups according to their use. As commercial customized supplements, some formulas are developed for a specific disease (kidney disease, intestinal disease, diabetes, congenital metabolic disease, dysphagia, etc.). As the market for whole foods has grown, the medical foods market has also grown steadily. As of 2019, the size of the medical foods market is estimated at USD 75 billion and grows by about 6% annually [12]. Recently, elderly people who require special nutrition, or who have difficulty chewing or swallowing, have also started using medical foods. The medical foods market is expected to expand further because the general public uses medical foods for nutritional supplementation.

Although many studies and new product development (NPD) projects have been conducted on the nutritional attributes of insects and their potential as medical foods, there are still few studies on the use of edible insects as medical foods from the consumer’s perspective or that reflect the hidden needs of them for medical foods. Most researchers avoid studies on why consumers choose insect-based food and how to promote it from the customer’s point of view; therefore, manufacturers start developing products that do not reflect the needs of the market, thereby pushing technology from the producer’s point of view. In general, there is an aversion to consuming insects [13], which leads to a decrease in the purchasing preference for edible insects. Producers are aware of this problem and have made various attempts to solve it. However, the attempted solutions were usually implemented from the producer’s point of view, not that of the consumer. At present, there is a need to form strategies from the consumer’s perspective that can actively overcome the aversions and promote the purchase of edible insects.

Based on the aforementioned information, this study aimed to help insect-based foods which had begun to be developed from the producer’s point of view to succeed without being disregarded by consumers. To this end, this study investigated the edible insect business and examined its problems using a questionnaire. This study presents medical foods made with edible insects (hereinafter referred to as insect medical foods) as one of the solutions for the edible insect business. The survey was conducted among caregivers, nurses, and doctors, who play a key role in providing medical foods to patients. This study is a descriptive study based on a survey that converged opinions on the ‘Innovation Community [14]’ around medical foods, which are currently neglected. These results are expected to contribute to increasing the likelihood of success for insect medical foods.

The remainder of the paper is organized as follows: Section 2 presents a theoretical background on medical and insect foods and the methodology of this study. Section 3 presents the results of the study, while these are extensively discussed in Section 4. Finally, Section 5 provides a summary of the implications of this study.

## 2. Materials and Methods 

### 2.1. Theoretical Background

#### 2.1.1. Characteristics of Medical Foods and Edible Insects

**Figure 1:**
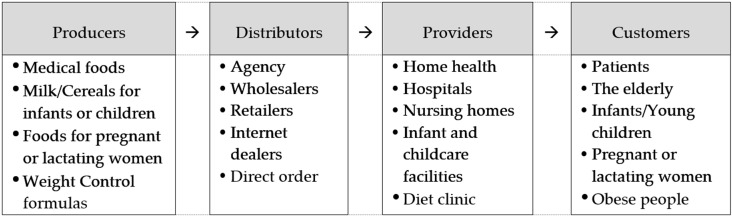
Industrial value chain of foods for special dietary uses.

Medical foods are classified as ‘foods for special dietary uses’ in Korea [15]. Foods for special dietary uses can be defined as food created for specific targets that require special nutritional management, such as infants/young children, patients, the elderly, the obese, or pregnant/lactating women. The industrial value chain of the food category is shown in Figure 1. Food producers distribute products through distributors, and distributors deliver products to customers through providers. Because of the nature of the product, some medical foods are delivered to consumers through hospitals or nursing homes. In the health care field where ‘information asymmetry’ is present [16], doctors and nurses who have more information and indirect experiences than patients have an important role in the industrial value chain of the foods.

Medical foods are formulated to provide general nutritional support to persons unable to ingest food in its conventional form, or to provide special nutritional support to persons with altered medical or physical conditions [4]. Some patients have limited ability to ingest, digest, or metabolize food; they have different physiological nutritional requirements to those of healthy people because of illness or other clinical conditions. Therefore, they should be provided with separate nutritional supplements instead of normal meals. Medical foods are intended to provide a balanced supply of nutrients, such as protein, fat, carbohydrates, etc. Each nutrient is composed of a variety of raw materials. Sodium casein and isolated soy protein are used as protein sources. The main fat ingredients are oils such as canola or soybean oil. Dextrin and fiber are used as raw carbohydrate sources. Some patients or their family make supplements at home by themselves or are supplied with them by the hospital. However, commercially developed medical foods are commonly used.

Currently, the medical foods available on the market have some limitations from the consumers’ perspective. First, medical foods do not fully satisfy the consumers’ needs to improve their health. It turns out that 69.7% of people who consume medical foods also consume other supplements, such as functional foods [17]. In the case of patients and elderly people, the main consumers of medical foods, there is a high demand for restoring health through the intake of dietary supplements, owing to limited diet and limited exercise. Nutritional intake is restricted by physicians because they do not know how food will affect the treatment and recovery from the disease. Therefore, medical foods are often the only food that patients can officially consume. Second, consumers have doubts about the stability of raw materials. Despite the recent increase in the consumption of processed foods, consumer doubts about the raw materials themselves are increasing. The Food Standards Agency [18], which has produced data on the degree of concern about food safety issues, presented the main consumer concerns as the following: chemicals (30%), food poisoning (29%), additives (29%), hormones/steroids/antibiotics (27%), and genetically modified organisms (GMOs) (24%). Many of these concerns are related to raw materials. 

Edible insects have the following nutritional and food safety benefits. First, edible insects are nutritionally superior and can satisfy consumers’ expectations. In terms of nutrition, edible insects can replace meat protein adequately, and they have the potential to serve as a major source of nutrition in medical foods. The nutrients of edible insects are equal or superior to beef, chicken, or pork [19,20,21]. Essential amino acids (EAAs) composition is an important indicator in food quality assessment. As shown in Table 1, the EAAs of edible insects are almost equivalent to those of chicken, beef, and pork.

Second, ingesting edible insects has been associated with various health benefits other than their nutritional value [2]. For example, caterpillar fungus is thought to have immunostimulant and anticancer effects [25]. Silkworms are effective in lowering blood glucose [26,27]; to such a high degree that they are being used to treat diabetes. In addition, the enzymatic hydrolysates of insects have antioxidant and antidiabetic effects, while they can also inhibit angiotensin-converting enzymes [27,28].

Finally, edible insects are safe, and users can consume them with confidence. The Ministry of Food and Drug Safety of Korea (MFDS) has designated seven types of insects that can be used as food. They are *Bombyx batryticatus*, *Bombyx mori L.*, *Oxya japonica Thungberg*, *Tenebrio molitor L.*, *Protaetia brevitarsis*, *Allomyrina dichotoma*, and *Gryllus bimaculatus* [14]. *Batryticatus Bombyx*, *Bombyx mori L.*, and *Oxya japonica Thungberg* have been traditionally used for food, and *Tenebrio molitor L.*, *Protaetia brevitarsis*, *Allomyrina dichotoma*, and *Gryllus bimaculatus* have been recognized as food sources since 2016. 

#### 2.1.2. The Necessity of User Innovation for Edible Insects to Become Medical Food

Open innovation refers to using the innovation achieved not only by utilizing the internal knowledge of the company, but also external knowledge, to create new markets and gain access to other business models or markets [29]. Open innovation provides a view of innovation, primarily from the company’s perspective. User innovation is an open innovation strategy in which consumers actively participate in the innovation process for products, services, and ideas provided by companies [30,31]. User innovation is compatible with open innovation because users can be regarded as a source of external innovation. Therefore, the perspective of early research on user innovation requires a user engagement method and is referred to as user-designed or having open innovation with customers.

The user innovation paradigm began with von Hippel’s research on user innovation with the concept of lead users. This was established through research on innovation strategy or policy [32]. Von Hippel showed that the manner with which lead users influence innovation and the pattern of user innovation differ from producer innovation. Lead users have real experience with a new product or process concept [33] and play the role of a need-forecasting laboratory [33,34,35]. However, in addition to creating innovative needs and ideas, lead users are considered a source of innovative solutions [32,34]. Von Hippel associated lead users with the concept of sticky information. This means that the needs of users are theoretical, thus making it difficult to communicate them effectively to a producer [36]. Successfully integrating lead users into the innovation process can overcome the stickiness of this information and solve its own functional fixation. Users have their own expertise and knowledge; hence, companies need to secure beneficial co-creation by matching phases, user roles, and user types for innovation projects [37]. 

Lettl et al. [38] presented an expansion model for user characteristics related to the types of tasks that users perform in the innovation process. Based on empirical research, they presented guidelines to recruit users for testing. Enkel et al. [39] and Jespersen [37] associated user types with different roles and steps in the NPD process; these types were lead users, first buyers, reference customers, requesting customers, and launching customers. These types of users are similar to what Grabher et al. [40] described as expressive users, who are motivated to share their experiences to contribute possible solutions that will ultimately lead to their needs being met more efficiently. Duverger and Hassan [41] suggest that unsatisfied users or users who have stopped using certain services or products (also called directors) are potential sources of innovative ideas. 

Kristensson et al. [42] concluded through empirical research that experiencing real situations has great implications for users when developing ideas for innovative NPD. Lettl et al. [38] mention some general challenges when involving users in radical innovation; for example, cognitive limitations can interfere with users’ ability to provide valuable input. Additionally, the users’ needs and demands tend to be theoretical and not obvious or practical [43,44]. In other words, many user needs are sticky [36,45], which means that it is difficult and expensive to communicate them to the producer. Many users naturally resist change or are not willing to contribute to fundamental innovation projects [46]. 

Various stakeholders are involved in healthcare innovation. Patients want to restore health. Healthcare professionals and caregivers provide healthcare services suitable for the patients. Companies seek profits by supplying medicine, medical foods, and medical devices to patients, caregivers, and healthcare professionals. According to Bjørkquist et al. [47], healthcare professionals and caregivers act as users and are expected to contribute to innovation by collaborating with developers based on asymmetric information. They participate in product innovation and development. In the study of Bullinger et al. [48], caregivers, healthcare employees, physicians, and family members participated in open innovation in healthcare. Burns [49] introduced a value chain for innovation in the healthcare business. It explained the existence of complex interactions among producers of healthcare products, providers of healthcare services, insurers, and healthcare consumers. In particular, in the flow of information, Burns [49] emphasized to accept the opinions of providers or consumers of healthcare services and reflect their innovations in products. Schiavone [50] classified ‘users’ of health technology into three main types: (1) healthcare professionals; (2) patients, their family members, and caregivers; and (3) other people involved in the healthcare industry. In this context, innovation by healthcare professionals and caregivers can be understood as a kind of user innovation. This user innovation helps producers to successfully launch new products by providing them with a lot of information about their needs [32]. It is expected that healthcare personnel with more information and indirect experiences of medical foods will provide more information toward user innovation.

### 2.2. Methodology

#### 2.2.1. Survey Structure

The survey contents of this study were determined from preliminary surveys of 15 medical service providers from 23 June to 9 July 2019. The preliminary survey was conducted to embody the questionnaire. The preliminary survey was about the basic perception of general food, medical food, and insect food. Respondents were concerned with food hygiene and safety. They suggested that the needs of various consumers should be reflected in the development of medical food. For insect food, it was found that the edible insect had a low profile, and it was important to overcome the aversion to it. It showed the possibility of success of various ideas to promote the insect medical food business. The questionnaire based on the preliminary survey consisted of four parts. Part 1 consisted of four questions about the demographic characteristics. The gender, age, role, and residence of the survey subjects were investigated. Part 2 consisted of seven questions about basic perception regarding food and edible insects. Three questions were presented on the consumption behavior of general processed foods and whether insect foods were consumed. As for the consumption of general processed foods, it is presented that one option be chosen among taste, quality, stability, price, and appearance. Respondents were asked to answer yes or no for the recognition of edible raw materials for insects, experience of processed edible insects for food, and whether or not to eat chrysalides. Part 3 consisted of seven questions about the reasons for providing insect medical foods to users. For the purpose of using medical food, one of four alternatives was selected. For the use of medical food, either ‘homemade’ or ‘bought processed food’ was selected. Respondents who answered yes or no were asked whether they had health trouble and whether they purchased insect medical food. The adequacy of the price was measured using the 5-point Likert scale. For the reasons for purchasing insect medical food, low price, good taste, environmental friendliness, new nutrition needs, and high nutrition value were presented, and a 5-point Likert scale was used for each item. Part 4 surveyed three questions about promotion strategies for insect medical foods. For promotion strategies, the following seven items were presented, and the effectiveness was measured using the 5-point Likert scale: price promotion, use of clean raw material, frequent exposure to media, disclosure of safety verification results, promotional campaign, frequent participation in events, and use of purified powder. The intention to change the purchase after promotional measures was measured using the 5-point Likert scale. In addition, the effectiveness of the government’s edible insect renaming policy was measured using the 5-point Likert scale.

#### 2.2.2. Data Collection

This study was conducted on healthcare professionals and caregivers because it was not appropriate to ask patients. They were working in four hospitals and four nursing homes that supply medical food to patients. This survey was conducted from 21 July to 26 September 2019. To randomly select the survey target hospitals, a total of four were selected from Seoul, Gyeonggido, Chungnam and Daejeon, and Chungbuk. In the same way, a total of four nursing homes were selected.

Fifteen questionnaires were sent to each of eight target institutions. It was approved in advance to conduct 15 surveys from each institutional review board (IRB). The questionnaire was sent to a total of 120 people. There were 114 respondents and six non-respondents; hence, the response rate was 95%. Among the completed questionnaires, 24 samples with insufficient responses were excluded. Ninety samples were used as the final analytical data for this study. A total of 90 participants (10 men and 80 women) were surveyed that belonged to different age groups; 14.4%, 27.8%, 32.2%, and 25.6% of the participants were in their 20s, 30s, 40s, and 50s, or older, respectively. The participants were classified according to their role: 18 caregivers, 65 nurses, and 7 doctors. Demographic characteristics are summarized in Table 2. Most of the respondents were nurses. However, nurses are creative and have excellent problem-solving skills because they solve the problems they encounter daily with patients [51]. Nurses can contribute to improving medical practices and the quality of care services and to advancing health policies and health information technologies [52]. If a comfortable, open, and caring relationship between the nurse and the patient is assured, it will help the patient to maximize the probability of sharing his or her ideas [50]. Nurses are innovative enough to lead user innovation in the medical food field. The study was conducted with IRB approval from each hospital and nursing home. To obtain IRB approval, investigators completed training from accredited bioethics educational institutions.

#### 2.2.3. Analytical Method

Factor analysis and reliability analysis were performed to verify the effectiveness of the promotional strategy by calculating the internal consistency of insect health food purchase or non-purchase reasons and Cronbach’s alpha (*ρ_T_*). *ρ_T_* is used to analyze the reliability of respondents to multiple items at a fixed time. *ρ_T_* is represented by Equation (1).

(1) $$\rho_{T}=\;\frac{k}{k-1}\;\left(1-\frac{\sum_{i-1}^{k}\sigma_{i}^{2}}{\sigma_{X}^{2}}\right)$$

In the test consisting of k items, the score and variance of the i-th item are as follows: *X*_*i*_ and $\sigma_{i}^{2}$. The sum of the scores of each item and its variance are as follows: $\overset{k}{\underset{i=1}{\sum }}X_{i}$ and $\sigma_{X}^{2}$.

This study also performed a one-way analysis of variance (ANOVA) to determine whether the means of key variables represent significant differences across groups of health care providers. One-way ANOVA is a statistical test method that compares means between three or more groups. The study was analyzed after recording and data cleanup using Statistical Package for Social Science (SPSS) Statistics, version 25.0 (IBM SPSS Statistics, Armonk, NY, USA). Frequency analysis was performed to analyze the demographic and purchasing characteristics of insect medical food.

## 3. Results

### 3.1. Results on Basic Perception of Food and Edible Insects

Seven questions were asked about the basic perception of food and edible insects. There are three basic questions about food and four questions about basic knowledge on edible insects.

This survey was conducted to investigate how the participants’ overall perception of taste affected their decision to purchase edible insect food. First, this study investigated the factors that are considered a top priority when purchasing processed foods. These factors, in descending order, were stability (32.2%), quality (32.2%), taste (24.4%), appearance (6.7%), and price (4.4%). 

The results of surveying participants’ attitudes toward new products on the market, showed that 46.7% of the respondents did not like trying new food products, 24.4% did, and 28.9% had a neutral attitude. These results show that healthcare professionals and caregivers have a rather conservative approach to food. Wilkinson et al. [53] confirmed that the reluctance to eat new foods and previous experience of consuming insects affect entomophagy.

Regarding the participants’ attitudes toward environmental consumerism, 50% of the respondents answered that they purchased food considering the environment (strongly agree 6.7%, agree 43.3%), while 32.2% had a neutral attitude to the topic. Only 17.8% of the respondents answered that they consumed food without any concern for the environment (strongly disagree 1.1%, disagree 16.7%). Based on the increased environmental awareness level of the participants, it could be suggested that they are not merely buying food, but they were also considering the environment.

The MFDS has approved the consumption of seven insects as food. When asked if they were aware of this, 18.9% of the participants answered positively and 81.1% answered negatively. This result suggests that many healthcare professionals and caregivers were unaware that there is a legal framework for the use of edible insects as food.

Recently, a variety of products such as cookies, puddings, and nutrition bars made from edible insects were released. When the participants were asked if they learned about this through the news or internet, 34.4% answered ‘no’ and 65.6% answered ‘yes’. This indicates that edible insects are not promoted adequately.

Chrysalides are served as a side dish in some traditional Korean restaurants. This study asked the participants if they ate chrysalides to assess if they consumed insects in their daily lives. Approximately twice as many answered ‘yes’ (67.8%) as answered ‘no’ (32.2%). Chrysalides tend to be perceived as just a side dish or snack unless they are greatly conscious. Additionally, their savory taste and outward appearance that is not overly abominable may facilitate their purchase as they tend to go unnoticed. 

When asked if edible insects are necessary for modern people, 42.2% of the participants responded that insects are necessary to secure future food safety, 17.8% answered that they needed it because they considered the environment, 51.1% would choose edible insects as a means of considering the next generation, and 27.8% answered that they bring new nutritional qualities to the table.

### 3.2. Reasons for Providing Insect Medical Foods to Users

Seven questions about the reasons for providing insect medical foods to patients were asked. There were four questions about the purchase of medical foods and three about the purchase of insect medical foods. 

When asked about the purpose of providing medical foods to patients, 34.9% of the participants answered that they used it as a nutritional supplement for elderly people, 30.2% as a nutritional supplement before and after surgery, 19.0% as a meal replacement, and 15.9% as part of a diet for special diseases such as diabetes.

The results showed that 75.7% of the participants provided commercially processed medical foods to patients and 24.3% provided homemade or hospital-made foods to patients. As it is inconvenient to prepare or cook food, family members and caregivers usually provide their patients with processed foods. 

When asked whether their patients had any health problems, such as diarrhea, abdominal pain, and allergies after eating medical foods, 81.9% of the respondents answered ‘no’ and 18.1% answered ‘yes’.

When asked about the price propriety of medical foods, 87.5% of the respondents answered, and reported that they are very expensive (51.6%) and expensive (35.9%). In particular, patients in long-term care and elderly people are expected to consume medical foods for a long time; hence, the financial burden is expected to be greater. 

When asked whether they would provide insect medical food for patients or not, 46.7% of the respondents said that they were willing to provide it for patients. Therefore, the success or failure of this business depends on persuading the 53.3% of respondents who would not provide it for their patients.

Exploratory factor analysis was conducted to verify the feasibility and reliability of the measurement items. For exploratory factor analysis, a factor extraction model for principal component analysis was selected, and analysis was performed through a Varimax orthogonal rotation method. In the factor extraction process, factor loading was higher than 0.5 and factorized only for factors with an eigenvalue greater than 1.0. In this study, it was found that the Kaiser–Meyer–Olkin (KMO) value was 0.732, which was acceptable. Bartlett’s sphericity test was found to be significant, and factor analysis was performed. *ρ_T_* was calculated for the reasons for purchase/non-purchase insect medical foods and effectiveness of promotion strategies. As shown in Table 3, the results were all higher than 0.7, which was judged to be good. Therefore, it was evaluated that there were no questions that interfered with reliability, and analysis was conducted without removing the items of the survey.

**Figure 2:**
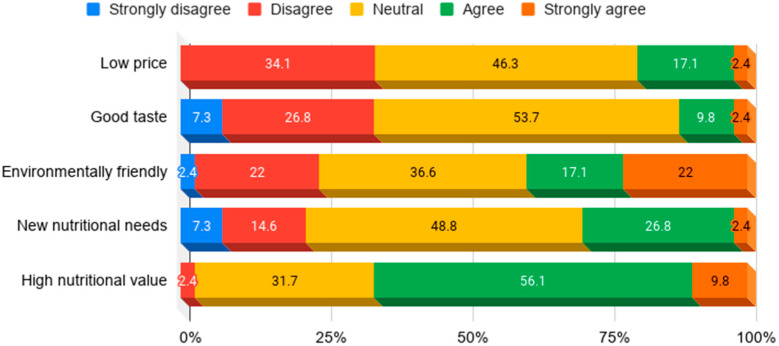
Reasons to supply insect medical foods to users.

Further, this study investigated the reasons why those willing to purchase insect medical foods would do so. Many respondents answered that this type of food is environmentally friendly, has high nutritional value, or fulfills new nutritional needs. Insects generally come across as environmentally friendly. Currently, most medical food products sold in the market are made from the same kind of material. Therefore, users are looking forward to the emergence of medical foods made from new sources. This study identified the need to find alternative materials to broaden users’ choices. There were only a few responses attributing the purchase of insect medical foods to their good taste or low price. The results are shown in Figure 2.

**Figure 3:**
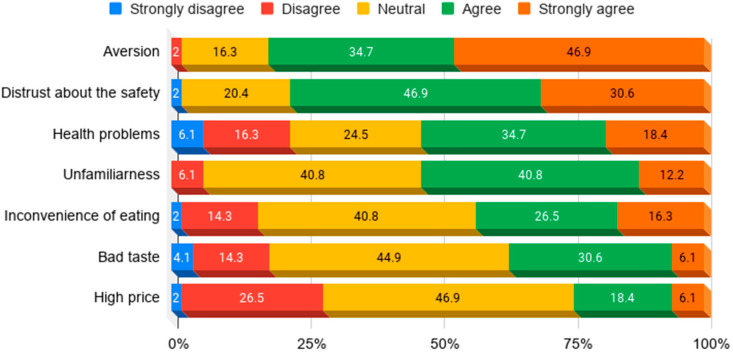
Reasons not to supply insect medical foods to users.

The reasons for not purchasing insect medical foods were also investigated. As is shown in Figure 3, the main causes were that participants were either disgusted by insects (strongly agree 46.9%, agree 34.7%) or overwhelmingly concerned about the safety of insects as raw materials (strongly agree 30.6%, agree 46.9%). Additionally, there were some concerns about health problems (strongly agree 18.4%, agree 34.7%). The results of the survey showed that the overall disgust and distrust of insects as raw materials were the biggest obstacles hindering the consumption of edible insects. Therefore, starting an insect medical food business could be difficult if the aversion to insects is not inversed and their safety as raw materials is not established.

### 3.3. Promotion Strategies for Insect Medical Foods

Three questions about how to promote the sale of insect medical food were presented. Seven ways to promote the sale of insect medical food were asked, and each effect was examined on the 5-point Likert scale. It was investigated whether the purchase intention was changed after implementing the promotion strategies. The effectiveness of the current government’s policy of “renaming edible insects” was also investigated.

**Figure 4:**
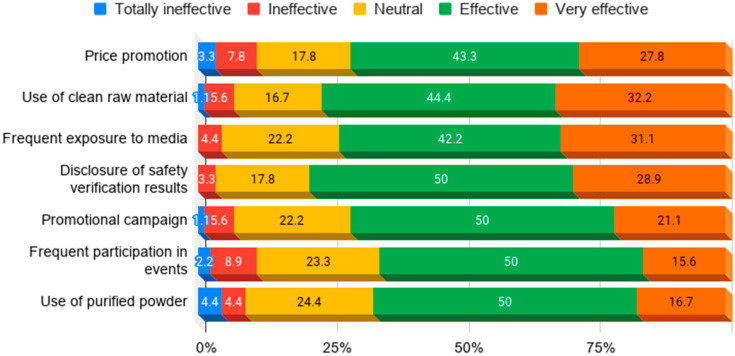
Efficiency of promotion strategies for insect medical foods.

When participants who had no intention of providing insect medical foods to patients were asked what measures would be effective in promoting the consumption of said foods, many answered that using clean raw materials, price promotions, and frequent media exposure would be effective measures (Figure 4). Many participants evaluated these strategies to be effective in increasing the attractiveness of insect medical foods.

One-way ANOVA was conducted to verify whether the means of the main variables showed significant differences according to the group of healthcare professionals and caregivers. As shown in Table 4, there was a significant difference between two promotion strategies depending on the group: ‘Use clean raw material’ (F = 4.012, *p* < 0.05) and ‘Frequent participation in events’ (F = 6.381, *p* < 0.01). Scheffe’s post-hoc analysis was performed on the variables with significant differences. As for the strategy “Use of clean raw material”, ‘d(doctor)’ was greater than ‘c(caregiver)’ and ‘n(nurse)’. As for the strategy “Frequent participation in events”, ‘c’ was greater than ‘n’ and ‘d’ was greater than ‘c’.

After implementing actions based on the users’ responses, we investigated whether they would be willing to purchase insect medical foods. When asked, 44.4% of the respondents said that they could change their decision (strongly agree 13.3%, agree 31.1%). Only 16.7% of the respondents said that they would not change their intention to purchase insect medical foods after the aforementioned actions had been implemented (strongly disagree 5.6%, disagree 11.1%), while 38.9% were neutral. In the end, incentive strategies are required to increase the purchasing power of the undecided class.

The government is looking into changing the name of edible insects to improve the consumers’ awareness of them. For example, the name of *Tenebrio molitor* has been changed to savory and that of *Protaetia brevitarsis* to flower beetle. We investigated whether these measures were helpful. When asked, 61.1% of respondents thought that this was a helpful action (very helpful 12.2%, helpful 48.9%), 25.6% had a neutral attitude, and 13.4% said that the actions did not help improve awareness (very unhelpful 5.6%, unhelpful 7.8%). Such awareness activities should be continuous if the image of insects is to be improved. If such activities are undertaken by national institutions, this could be helpful in promoting edible insect businesses.

## 4. Discussion

Morrison, Roberts, and von Hippel [54] studied user innovation led by the ‘library staff’ located between ‘users’ and ‘program developers’ in a study of the Australian Library’s Online Public Access System (OPAC). In the middle of the service supply chain, they achieved performances from user innovation. In the healthcare field, healthcare professionals and caregivers have traditionally been engaged in user innovation. Omachonu and Einspruch [55] showed that the innovation process is proceeded by providing feedback from healthcare professionals. In this context, medical food providers can contribute to the user innovation of the medical food business. 

Furthermore, the discussion about user in healthcare industry is expanding from healthcare professionals and caregivers to patients. Patient innovation improves the patient’s treatment, treatment experience, and treatment compliance [56]. It also reduces treatment costs. Therefore, it can be argued that patient-driven innovation in the healthcare field is the key to user innovation. However, user innovation in healthcare innovation does not mean only patient-driven innovation. Patient-driven innovation is a new concept that has only attracted researchers from developed countries in recent years. Patient innovation is defined as the modification or development of treatment, behavioral strategies, technical assistance, or medical devices for patients or non-professional caregivers to cope with the disease [57]. Many definitions are currently being used to define patient innovation, which is a new concern for healthcare management; hence, research is still ongoing [50]. Patient innovation activities and health care professional activities are complementary rather than competitive activities [58].

It was found that 18.1% of patients who ate the existing medical food had health trouble. Health abnormalities in the patient’s digestive system are likely to result from food allergy. Food allergy is an abnormal adverse reaction caused by a specific immune response after eating certain foods [59]. The prevalence of food allergies in Asia, Korea [60], Japan [61], Hong Kong [62], and Taiwan [63] ranged from 3.4 to 7%. Compared to the results of previous studies, the prevalence investigated in this study is more than twice the prevalence of common food allergies. This is one of the reasons why medical food users should have more options for food free from food allergies.

Entomophagy is influenced by people’s history, habits, traditions, and religious beliefs, as well as the delivery route of information about insects, rather than the degree of economic development [64]. To popularize entomophagy, this study considered medical foods and derived the two most effective alternatives from the healthcare professionals and caregivers’ perspective: participate in insect-related events frequently and use clean raw materials. First, edible insect-promoting activities should be publicized by frequently holding insect-related events. In fact, the Korean government supports various insect-related events to encourage the insect farming industry. Such events help to improve the consumers’ awareness of insects. Academic symposiums in particular can enhance consumers’ confidence by providing scientific information about edible insects. This could be an effective means of persuading consumers who eat medical foods but worry about health problems associated with the consumption of edible insects to consume the latter. The results of an empirical study by Liu, Li, and Gómez [65] confirmed that educating consumers and increasing their knowledge on edible insects increases their likeliness of purchasing them. Belgium is actually one of the most popular countries for edible insects. In a study of Belgium, researchers suggest that consumers prefer to buy it in a supermarket than at an event [66]. However, Korea is still less popularized for edible insects; hence, it seems to be trying to accumulate information through events and explore it.

Second, consumers were also interested in clean ingredients. Edible insects for food should be clean and wholesome (free from foreign materials, pathogens, toxins, etc.); produced, packaged, stored, and transported under hygienic conditions; and properly labeled [67]. It is expected that supporting the breeding of edible insects in clean areas would be effective. It should be noted that consumers trust domestic raw materials. Therefore, producing edible insects in a domestic clean area could be a successful strategy. Insects do not pollute the soil, rivers, or the air, therefore the areas they will be farmed in will remain clean. Consequently, these insect characteristics can help to boost consumers’ confidence regarding the safety of insects as clean raw materials. In addition, insect medical food producers should inform consumers that there is no question about the use of safe ingredients. Consumers have the preconceived notion that the insects’ breeding environment, breeding methods, and distribution environment will not be clean because they are not familiar with the material itself. Insects are more advantageous than meat or other crops because, by nature, they are free from pesticides and antibiotics in the breeding stage and their waste is easy to handle. 

Despite providing various contributions to edible insect food and medical food, this study has the following limitations. First, the sample size is small. The participants were healthcare professionals and caregivers. In the healthcare section, the healthcare professionals and caregivers have a lot of information and indirect experience in related fields; hence, this study pursues user innovation by conveying their reliable information to product developers. However, the failure to collect the same number of samples from each class of the healthcare section could have biased the results. This study could not recruit equal samples by gender or role. Samples were not recruited through sufficient prior consultation with hospital officials. Second, despite the fact that healthcare professionals and caregivers have a lot of information and experience, their answers may be biased depending on the patients they care for or the information and experience they have. Third, the lack of prototypes is another limitation of this study. In the NPD project, according to the user innovation perspective, it is important to quickly create and provide product prototypes to consumers based on the elements most needed by users [68]. Users who receive these prototypes will use it and repeatedly give feedback. If the survey is conducted without a prototype, the participants may have difficulty understanding the questions. These limitations will be complemented by future studies to enlarge the sample sizes, collaborate with physicians, recruit the same numbers of samples from each role, and use prototypes.

## 5. Conclusions

Existing medical foods do not fully reflect the consumers’ need to improve the health of patients and the elderly. As an alternative, this study suggested medical foods made from edible insects. An aversion to insects was confirmed as the most serious problem when using insect medical foods. Edible insects were recognized by participants as novel promising future food sources, while also being environmentally friendly. However, some participants responded negatively to consuming insect medical foods, as they expressed their aversion to insects as raw materials and their distrust regarding their safety. Additionally, some participants were concerned about health problems after eating insects. Nevertheless, companies that produce insect medical foods were evaluated to be effective in using clean raw materials and holding insect-related events frequently. Frequent media exposure or price promotion has not been proven effective to promote insect medical foods. Government support or cooperation with farmers’ groups is necessary to obtain clean raw materials or to hold related events. Moreover, there is a need to introduce a quality assurance system that will verify the hygiene and safety conditions of breeding insects.

This study provides two implications for the edible insect food industry. First, a strategy for developing insect food is presented from the healthcare professionals and caregivers’ perspective. As the edible insect business has developed, there have been many proposals for insect medical foods. However, most studies have been proposed from the producer’s point of view. Their argument is that insects have many health benefits, so that consumers should buy them. This study approached the topic from a healthcare professionals and caregivers’ perspective. That is why there are keys for entrepreneurs who have vaguely started in the insect medical food business and have reached the limit of problem solving from the voice of consumers. Second, insights by healthcare professionals and caregivers are also presented for medical food. This study asked participants why they would need to provide insect medical foods to users. In addition, this study helped participants to produce a way of self-promoting insect medical foods. Eventually, the results of this study contribute to the establishment of a win–win strategy that overcomes the limitations of existing medical food businesses and makes a breakthrough in the edible insect business.

## Figures and Tables

**Table 1:** Essential amino acid content of edible insects (based on dry matter, g/kg).

Animal	His	Ile	Leu	Lys	Met	Phe	Thr	Trp	Val
Mealworm (*Tenebrio molitor*)	8.3	13.1	22.0	15.8	6.0	13.0	12.6	2.9	18.9
Silkworm, larvae (*Bombyx mori*)	6.8	9.5	17.0	16.9	7.7	11.5	11.0	3.4	13.6
Cricket, adult (*Acheta domesticus*)	6.9	10.9	23.4	17.3	4.4	10.3	12.0	2.9	20.1
Chicken (breast, skinless)	8.3	11.0	18.6	21.6	5.8	9.0	10.0	2.8	11.6
Beef (steak, lean and fat)	6.7	9.6	16.8	17.9	5.5	8.3	8.4	1.3	10.5
Pork (leg and lean, raw)	8.1	9.5	16.4	18.4	5.4	8.1	9.3	2.6	11.1

(Source: Köhler et al. [22]; Wu et al. [23]; USDA database [24]).

**Table 2:** Demographic characteristics.

Variables	Classification	Number	Frequency (%)
Gender	Men	10	11.1
	Women	80	88.9
Age	20s	13	14.4
	30s	25	27.8
	40s	29	32.2
	≥50s	23	25.6
Role	Caregivers	18	20.0
	Nurses	65	72.2
	Doctors	7	7.8
Residence	Seoul	35	38.9
	Gyeonggido	25	27.8
	Chungnam & Daejeon	13	14.4
	Chungbuk	17	18.9

**Table 3:** Reliability analysis of measurement tools.

Variables	Cronbach’s Alpha	Item
Reason for purchase	0.747	5
Reason for non-purchase	0.821	7
Efficiency of promotion	0.759	7

**Table 4:** Differences in main variables according to healthcare professionals and caregivers.

Dependent Variables	Group	N	Mean	SD	*F*	*p*	Scheffe
1. Price promotion	caregiver (c)	18	2.00	0.90	0.499	0.609	-
	nurse (n)	65	2.12	0.94			
	doctor (d)	7	2.43	1.27			
2. Use of Clean raw material	caregiver (c)	18	2.00	0.97	4.012 *	0.022	c, n < d
	nurse (n)	65	2.00	0.81			
	doctor (d)	7	3.00	1.41			
3. Frequent exposure to media	caregiver (c)	18	2.00	0.97	0.845	0.433	-
	nurse (n)	65	1.98	0.83			
	doctor (d)	7	2.43	0.78			
4. Disclosure of safety verification results	caregiver (c)	18	2.06	0.93	0.061	0.941	-
	nurse (n)	65	2.26	2.60			
	doctor (d)	7	2.14	0.69			
5. Promotional campaign	caregiver (c)	18	2.17	0.85	0.749	0.476	-
	nurse (n)	65	2.15	0.85			
	doctor (d)	7	2.57	0.97			
6. Frequent participation in events	caregiver (c)	18	2.44	1.04	6.381 **	0.003	n < c < d
	nurse (n)	65	2.20	0.79			
	doctor (d)	7	3.43	1.13			
7. Use of purified powder	caregiver (c)	18	2.17	0.98	1.403	0.251	-
	nurse (n)	65	2.28	0.85			
	doctor (d)	7	2.86	1.57			

* *p* < 0.05, ** *p* < 0.01.

## References

[1] Han R., Shin J.T., Kim J., Choi Y.S., Kim Y.W. (2017). An overview of the South Korean edible insect food industry: Challenges and future pricing/promotion strategies. Entomol. Res..

[2] Raheem D., Carrascosa C., Oluwole O.B., Nieuwland M., Saraiva A., Millán R., Raposo A. (2019). Traditional consumption of and rearing edible insects in Africa, Asia and Europe. Crit. Rev. Food Sci. Nutr..

[3] Van Huis A., Van Itterbeeck J., Klunder H., Mertens E., Halloran A., Muir G., Vantomme P. (2013). Edible Insects: Future Prospects for Food and Feed Security.

[4] Raubicheck C.J. (1989). Labeling a medical food. Food Drug Cosm. Lj.

[5] Murefu T.R., Macheka L., Musundire R., Manditsera F.A. (2019). Safety of wild harvested and reared edible insects: A review. Food Control.

[6] Dobermann D., Swift J.A., Field L.M. (2017). Opportunities and hurdles of edible insects for food and feed. Nutr. Bull..

[7] DeFoliart G.R. Insects as a Global Food Resource: The History of Talking about It at the University of Wisconsin. https://insectsasfood.russell.wisc.edu/wp-content/uploads/sites/246/2012/09/Manuscript.pdf.

[8] Yen A.L. (2015). Conservation of Lepidoptera used as human food and medicine. Curr. Opin. Insect Sci..

[9] Williams D.D., Williams S.S. (2017). Aquatic insects and their potential to contribute to the diet of the globally expanding human population. Insects.

[10] Yun E., Hwang J. (2016). Status and prospect for development of insect foods. Food Sci. Ind..

[11] Kim H. Patient food development with edible insects. Proceedings of the International Symposium for Edible Insect Industry, Rural Development Administration.

[12] Li L. (2019). Study on the Application of Dietary Fiber on Special Medicine. Int. J. New Dev. Eng. Soc..

[13] Looy H., Dunkel F.V., Wood J.R. (2014). How then shall we eat? Insect-eating attitudes and sustainable foodways. Agric. Hum. Values.

[14] Fichter K. (2009). Innovation communities: The role of networks of promotors in Open Innovation. Rd Manag..

[15] Ministry of Food and Drug Safety Food Code. https://www.foodsafetykorea.go.kr/foodcode.

[16] Bloom G., Standing H., Lloyd R. (2008). Markets, information asymmetry and health care: Towards new social contracts. Soc. Sci. Med..

[17] Korea Agro-Fisheries & Food Trade Corporation (2016). 2015 Processed Food Submarket Report-Medical Food.

[18] Food Standards Agency (2019). Public Attitudes Tracker. https://www.food.gov.uk/sites/default/files/media/document/public-attitudes-tracker-wave-18-final_0.pdf.

[19] Akhtar Y., Isman M.B. (2018). Insects as an Alternative Protein Source. Proteins in Food Processing.

[20] Fombong F.T., Van Der Borght M., Vanden Broeck J. (2017). Influence of freeze-drying and oven-drying post blanching on the nutrient composition of the edible insect Ruspolia differens. Insects.

[21] Finke M.D. (2002). Complete nutrient composition of commercially raised invertebrates used as food for insectivores. Zoo Biol. Publ. Affil. Am. Zoo Aquar. Assoc..

[22] Köhler R., Kariuki L., Lambert C., Biesalski H.K. (2019). Protein, amino acid and mineral composition of some edible insects from Thailand. J. Asia-Pac. Entomol..

[23] Wu R.A., Ding Q., Yin L., Chi X., Sun N., He R., Luo L., Ma H., Li Z. (2020). Comparison of the nutritional value of mysore thorn borer (Anoplophora chinensis) and mealworm larva (Tenebrio molitor): Amino acid, fatty acid, and element profiles. Food Chem..

[24] (2019). USDA Database. https://fdc.nal.usda.gov.

[25] Feng Y., Zhao M., He Z., Chen Z., Sun L. (2009). Research and utilization of medicinal insects in China. Entomol. Res..

[26] Belluco S., Losasso C., Maggioletti M., Alonzi C.C., Paoletti M.G., Ricci A. (2013). Edible insects in a food safety and nutritional perspective: A critical review. Compr. Rev. Food Sci. Food Saf..

[27] Nongonierma A.B., FitzGerald R.J. (2017). Unlocking the biological potential of proteins from edible insects through enzymatic hydrolysis: A review. Innov. Food Sci. Emerg. Technol..

[28] De Castro R.J.S., Ohara A., dos Santos Aguilar J.G., Domingues M.A.F. (2018). Nutritional, functional and biological properties of insect proteins: Processes for obtaining, consumption and future challenges. Trends Food Sci. Technol..

[29] Chesbrough H. (2004). Managing open innovation. Res. Technol. Manag..

[30] Di Gangi P.M., Wasko M. (2009). Steal my idea! Organizational adoption of user innovations from a user innovation community: A case study of Dell IdeaStorm. Decis. Support Syst..

[31] Faulkner P., Runde J. (2009). On the identity of technological objects and user innovations in function. Acad. Manag. Rev..

[32] Von Hippel E. (2005). Democratizing innovation: The evolving phenomenon of user innovation. J. Für Betr..

[33] Von Hippel E. (1986). Lead users: A source of novel product concepts. Manag. Sci..

[34] Lilien G.L., Morrison P.D., Searls K., Sonnack M., Hippel E.V. (2002). Performance assessment of the lead user idea-generation process for new product development. Manag. Sci..

[35] Lüthje C., Herstatt C. (2004). The Lead User method: An outline of empirical findings and issues for future research. Rd Manag..

[36] Von Hippel E. (1994). “Sticky information” and the locus of problem solving: Implications for innovation. Manag. Sci..

[37] Jespersen K.R. (2008). User Driven Product Development: Creating a User-Involving Culture.

[38] Lettl C., Herstatt C., Gemünden H.G. (2006). Learning from Users for Radical Innovations. Int. J. Technol. Manag..

[39] Enkel E., Perez-Freije J., Gassmann O. (2005). Minimizing market risks through customer integration in new product development: Learning from bad practice. Creat. Innov. Manag..

[40] Grabher G., Ibert O., Flohr S. (2008). The neglected king: The customer in the new knowledge ecology of innovation. Econ. Geogr..

[41] Duverger P., Hassan S.S. (2008). Defectors as a new source of radical service innovation ideas. White Pap..

[42] Kristensson P., Matthing J., Johansson N. (2008). Key strategies for the successful involvement of customers in the co-creation of new technology-based services. Int. J. Serv. Ind. Manag..

[43] Sowter C.V. (2018). Marketing High Technology Services.

[44] Franke N., Schreier M. (2002). Entrepreneurial opportunities with toolkits for user innovation and design. Int. J. Media Manag..

[45] Ogawa S. (1998). Does sticky information affect the locus of innovation? Evidence from the Japanese convenience-store industry. Res. Policy.

[46] Lettl C. (2007). User involvement competence for radical innovation. J. Eng. Technol. Manag..

[47] Bjørkquist C., Ramsdal H., Ramsdal K. (2015). User participation and stakeholder involvement in health care innovation–does it matter?. Eur. J. Innov. Manag..

[48] Bullinger A.C., Rass M., Adamczyk S., Moeslein K.M., Sohn S. (2012). Open innovation in health care: Analysis of an open health platform. Health Policy.

[49] Burns L.R. (2012). The Business of Healthcare Innovation.

[50] Schiavone F. (2020). User Innovation in Healthcare. User Innovation in Healthcare.

[51] Bratton B. (2017). Nurses as Innovators. J. Pediatric Surg. Nurs..

[52] Thomas T.W., Seifert P.C., Joyner J.C. (2016). Registered Nurses Leading Innovative Changes. Ojin Online J. Issues Nurs..

[53] Wilkinson K., Muhlhausler B., Motley C., Crump A., Bray H., Ankeny R. (2018). Australian consumers’ awareness and acceptance of insects as food. Insects.

[54] Morrison P.D., Roberts J.H., Von Hippel E. (2000). Determinants of user innovation and innovation sharing in a local market. Manag. Sci..

[55] Omachonu V.K., Einspruch N.G. (2010). Innovation in healthcare delivery systems: A conceptual framework. Innov. J. Public Sect. Innov. J..

[56] Barello S., Graffigna G., Vegni E. (2012). Patient Engagement as an Emerging Challenge for Healthcare Services: Mapping the Literature. Nurs. Res. Pr..

[57] Habicht H., Oliveira P., Shcherbatiuk V. (2013). User Innovators: When Patients Set Out to Help Themselves and End Up Helping Many. Die Unternehm..

[58] Zejnilović L., Oliveira P., Canhão H. (2016). Innovations by and for patients, and their place in the future health care system. Boundaryless Hospital.

[59] Wu T., Tsai T., Huang C., Chang F., Lin C., Huang I., Chu C., Lau B., Wu L., Peng H. (2012). Prevalence of Food Allergy in T Aiwan: A Questionnaire-based Survey. Int. Med. J..

[60] Ho M.H., Lee S.L., Wong W.H., Patrick I.P., Lau Y.L. (2012). Prevalence of Self-Reported Food Allergy in Hong Kong Children and Teens-a Population Survey. Asian Pac. J. Allergy Immunol..

[61] Leung T.F., Yung E., Wong Y.S., Lam C.W., Wong G.W. (2009). Parent-reported Adverse Food Reactions in Hong Kong Chinese pre-schoolers: Epidemiology, Clinical Spectrum and Risk Factors. Pediatric Allergy Immunol..

[62] Kusunoki T., Morimoto T., Nishikomori R., Heike T., Fujii T., Nakahata T. (2009). Allergic Status of Schoolchildren with Food Allergy to Eggs, Milk Or Wheat in Infancy. Pediatric Allergy Immunol..

[63] Kim J., Chang E., Han Y., Ahn K., Lee S. (2011). The Incidence and Risk Factors of Immediate Type Food Allergy during the First Year of Life in Korean Infants: A Birth Cohort Study. Pediatric Allergy Immunol..

[64] Ghosh S., Jung C., Meyer-Rochow V.B., Dekebo A. (2020). Perception of entomophagy by residents of Korea and Ethiopia revealed through structured questionnaire. J. Insects Food Feed..

[65] Liu A., Li J., Gómez M.I. (2020). Factors Influencing Consumption of Edible Insects for Chinese Consumers. Insects.

[66] Van Thielen L., Vermuyten S., Storms B., Rumpold B., Van Campenhout L. (2019). Consumer acceptance of foods containing edible insects in Belgium two years after their introduction to the market. J. Insects Food Feed..

[67] Van Huis A. (2016). Edible insects are the future?. Proc. Nutr. Soc..

[68] Martin J. (1991). Rapid Application Development.

